# Development of a new COVID-19 panel survey: the IAB high-frequency online personal panel (HOPP)

**DOI:** 10.1186/s12651-021-00295-z

**Published:** 2021-06-23

**Authors:** Georg-Christoph Haas, Bettina Müller, Christopher Osiander, Julia Schmidtke, Annette Trahms, Marieke Volkert, Stefan Zins

**Affiliations:** 1 Institute for Employment Research, Nuremberg, Germany; 2 University of Mannheim, Mannheim, Germany

## Abstract

Since January 2020, the COVID-19 crisis has affected everyday life around the world, and rigorous government lockdown restrictions have been implemented to prevent the further spread of the pandemic. The consequences of the corona crisis and the associated lockdown policies for public health, social life, and the economy are vast. In view of the rapidly changing situation during this crisis, policymakers require timely data and research results that allow for informed decisions. Addressing the requirement for adequate databases to assess people’s  life and work situations during the pandemic, the Institute for Employment Research (IAB) developed the High-frequency Online Personal Panel (HOPP). The HOPP study started in May 2020 and is based on a random sample of individuals drawn from the administrative data of the Federal Employment Agency in Germany, containing information on all labour market participants except civil servants and self-employed. The main goal of the HOPP study is to assess the short-term as well as long-term changes in people’s social life and working situation in Germany due to the corona pandemic. To assess individual dynamics the HOPP collected data on a monthly (wave  one to four) and bi-monthly (wave five to seven) basis. Furthermore, respondents were divided into four groups. The different groups of a new wave were invited to the survey at weekly intervals (wave two to four) or bi-weekly intervals (wave five to seven). This gives us the advantage of being able to provide weekly data while each participant only had to participate on a monthly or bi-monthly basis. In this article, we delineate the HOPP study in terms of its main goals and features, topics, and survey design. Furthermore, we provide a summary of results derived from HOPP and the future prospects of the study.

## Introduction

Since January 2020, the COVID-19 crisis has affected everyday life around the world, and rigorous government lockdown restrictions have been implemented to prevent the further spread of the pandemic. The consequences of the corona crisis and the associated lockdown policies for public health, social life, and the economy are vast. In view of the rapidly changing situation during this crisis, policymakers require timely data and research results that allow for informed decisions.

Addressing the demand for adequate databases to assess people’s life and work situations during the pandemic, the Institute for Employment Research (IAB) developed the High-frequency Online Personal Panel (HOPP), which started in May 2020. The HOPP study was designed to flexibly capture short-term individual dynamics in the labour market and labour market-related elements as the COVID-19 crisis unfolds. In addition, long-term effects can be evaluated by linking administrative process data from the Federal Employment Agency (FEA), the Integrated Employment Biographies (IEB) (Jacobebbinghaus and Seth 2007).

In the following, we delineate the HOPP study in terms of its main goals and features, topics, and survey design. Furthermore, we provide a summary of results derived from HOPP and the future prospects of the study.

## Substantive goals and features of the HOPP study

The HOPP study was initiated to evaluate how the corona crisis is affecting individuals in the German labour market. To obtain a complete picture of people’s  life and work situations during the pandemic, HOPP was designed to flexibly address new topics as the crisis evolves. The questionnaire therefore contains a mix of core modules on employment and labour market-related aspects of life as well as questions and modules that can be introduced depending on situational changes due to lockdown measures, e.g., regarding short-time work, organization of childcare, home office, health, and attitudes (Sect. 3).

Apart from this substantive aim, the HOPP has three distinct methodological features that set it apart from other corona-related panel studies: a probability sample design, high-frequency data collection, and linkage with administrative data.

*Probability sample design*: To adequately represent individuals in the German labour market, the HOPP study is based on a random sample of individuals drawn from the IEB (see Sect. 4.1). This gives it a major advantage over most of the online surveys implemented to evaluate the impact of the corona crisis, as the latter are based primarily on online convenience samples and therefore lack generalizability due to selection bias (see Schaurer & Weiß 2020).

*High-frequency data collection*: As decisions during the corona crisis have to be made very quickly, the survey period and frequency of data collection are crucial to informing such decisions. To closely monitor individual dynamics and to address newly arising data demands in as timely a manner as possible, the HOPP study collected data monthly (waves one to four) and bi-monthly (five to seven). Furthermore, to monitor changes on a weekly basis, the sample was divided into four groups of respondents who were surveyed at one-week intervals (see Sect. 4.3).

*Linkage with administrative data*: Another feature of the HOPP study is that survey data can be supplemented with administrative data from the FEA including information on employment spells for all employment that is subject to social security, benefit receipt, job searches, and participation in employment and training measures (Sect. 6). Linked with administrative data, the HOPP study can serve as a database to evaluate the long-term effects of the corona crisis on employment.

## Topics and questionnaires

The HOPP study collects data on the current employment situation and labour market-related aspects of individuals in Germany. Specifically, the main questionnaire programme through wave seven includes topics concerning employment, subsidized short-term work, childcare, home office, life satisfaction, and health. In addition, focus topics address couples’ division of childcare and housework before and during the corona crisis (wave two), vocational training (wave three), experiences with home office and reasons for not working from home (wave four), work-life balance (wave five), abuse of legal provisions regarding short-time work (waves six and seven), and trust in institutions and democracy (wave seven). Appendix Table 2 provides an overview of the variables in waves one to seven.

Although based on a sample of individuals, the HOPP study addresses labour market-related topics in the context of households (e.g., childcare). Therefore, we collected several household characteristics, e.g., the household composition, the number of children aged 18 or younger living in the household, and the children’s date of birth. Furthermore, respondents who report being in a relationship are asked to provide information on their partner’s current employment status, short-time work, and working hours and whether and to what extent their partner works from home.

The questionnaire modules were developed primarily by the Institute for Employment Research and in cooperation with external researchers. The questionnaires also contain items from other studies, namely, the German Internet Panel (GIP),^1^ the German Family Panel pairfam,^2^ and the German Socioeconomic Panel (GSOEP).^3^ For a comprehensive list of items and references for the items that were taken or adapted from other studies, please refer to the HOPP Codebooks and the Data Manual.^4^

## Study design

**Fig. 1:**
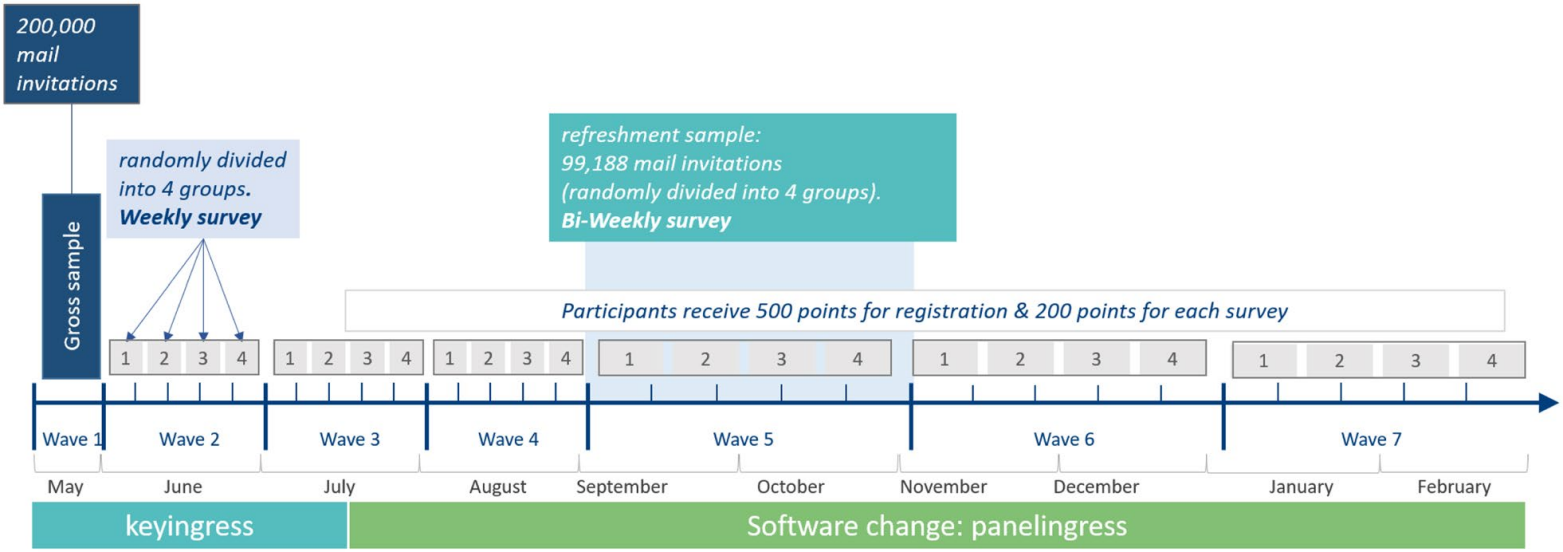
Structure of the study

In the following, we describe the study design with respect to sampling, the panel recruitment and contact strategy, the frequency of data collection, panel maintenance and incentives and show how response rates developed over time. Given the rapid setup of the HOPP study, some features of the panel were introduced in later waves (e.g., incentives, panel software) or modified over the course of the study (frequency of data collection). These changes are displayed in Fig. 1, together with a description of the respective design features.

### Sampling design

The sample for the HOPP study was drawn from the IEB. The IEB contains administrative labour market records that employers, job centres and employment agencies report to the federal employment agency in Germany. These records contain all individuals who have at least one of the following spells^5^: employment subject to social security, marginal part-time employment, receipt of benefits, participation in an employment or training measure or registration as a jobseeker at the Federal Employment Agency (e.g., see Antoni et al. 2019). This excludes individuals within the labour force who are civil servants or self-employed.

The sampling frame for the HOPP considers IEB reports with a reporting date until December 31st, 2018, and limits reports to all individuals who reached their 18th year on May 1st, 2020, or before and had at least one data entry report in 2018. The IEB can be linked to individual contact data (name and postal address), which allows individuals to be sent an invitation letter by mail to participate in an online survey.

A stratified sample with simple random sampling within strata was used, with strata defined by region, age, gender, and employment status in 2018. Specifically, administrative units, called regional directorates (Regionaldirektionen), of the Federal Employment Agency were used for geographical stratification. Age on May 1st, 2020, was categorized as 18–29, 30–39, 40–49, 50–59, and 60–99. The strata on employment status in 2018 include four categories: (a) individuals who had only employment spells in 2018, with at least one spell of marginal employment, (b) individuals who had only employment spells in 2018, with no spell of marginal employment, (c) individuals who received unemployment benefit II (means-tested basic income for jobseekers) at least once in 2018, and (d) individuals who did not receive unemployment benefit II in 2018 but were at least once registered at the Federal Employment Agency for other reasons (receipt of unemployment benefit from the unemployment insurance system, participation in a measure of active labor market policies, registration as jobseeker).

The gross sample size was allocated proportionally to the total number of persons within the respective strata of the sampling frame, i.e., inclusion probabilities were equal for all persons in the sampling frame (0.0043). The exceptions to this rule were older employees in the 60–99 age group who were employed in 2018 and marginal part-time employees who had a higher sampling fraction (0.0063) than persons in the other strata. A higher sampling fraction for those groups was chosen to address research questions requiring a higher number of respondents.

As the distribution of stratification variables is mostly proportional to their distribution within the IEB sampling frame, the share of persons in our sample that belong to strata with unemployed persons or welfare recipients is relatively small. This diminishes the statistical power of any analysis that is specific to one of these subgroups, compared to employees. With regard to inferential statistics, it is therefore recommended that analyses be conducted either for the whole German labour market or employed individuals only.

Panel participants were recruited at two times: during wave one in May 2020 and during wave five in September/October 2020. The net sample was defined to contain approximately 10,000 complete interviews for wave one. Judging from other studies at the IAB with a similar target population, mode, and sampling and contact strategy, we expected a response rate of approximately five percent. Therefore, a sample of 200,000 was selected from our IEB sampling frame. For wave five, we selected a refreshment sample of 99,188 cases with the same design.

### Panel recruitment and contact strategy

We recruited respondents by sending them an invitation by mail on May 8th, 2020. The letter contained information about the objectives of the study, information on data protection regulations, a short URL link to the online survey, an individualized randomly generated password to access the survey^6^ and a QR code to facilitate participation via smartphone. In addition, a URL link to a homepage providing more detailed information, e.g., on data protection, was included.

At the end of wave one, respondents were asked for their consent to be contacted for follow-up waves. The design of the HOPP study is non-monotonic, that is, respondents who did not participate in a given wave are invited to the next wave, provided panel consent was given in the initial interview.

**Fig. 2:**
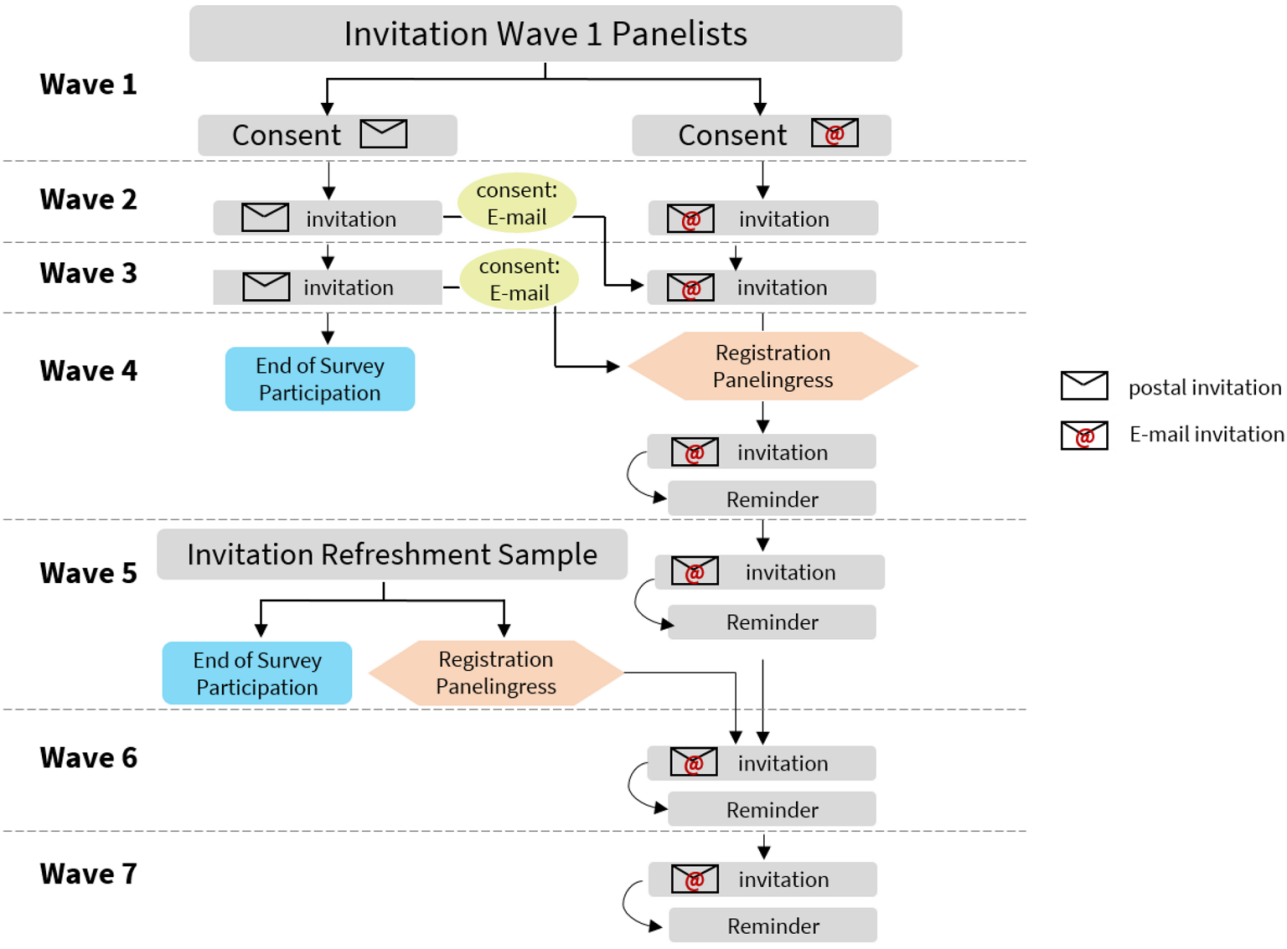
Flowchart showing all of the recruitment, maintenance, registration steps

To simplify the field work, panellists were moved to a panel website during the data collection in waves three and four and had to register themselves (see Panel Maintenance and Incentives for more details). To save resources, we stopped contacting respondents who did not register themselves on the panel website from wave five on (Fig. 2).

In wave five, we invited a refreshment sample by mailing them an invitation letter similar to the one in wave one. At the end of wave five, refreshment respondents were asked for their consent to be contacted for follow-up waves. If respondents provided their consent for re-contact, we asked respondents to register themselves on the panel website to download their promised incentive and to be invited to follow-up waves. In contrast to wave one, we did not ask for consent to contact respondents who did not provide an e-mail address with a postal letter in subsequent waves, to reduce field management costs.

In the first five waves, we invited panellists on Fridays (letters were mailed on Thursdays). From wave six on, we changed the invitation day to Monday, as we expected higher response rates by inviting people at the beginning of a week based on findings from other studies (Lindgren et al. 2020; Blom et al. 2020).

### Frequency of data collection

To monitor changes during the COVID-19 pandemic, a high frequency of data collection was needed. To meet this demand, we divided the frequency of data collection into two levels and adjusted the frequency of data collection over time (see Fig. 1). The first level of data collection frequency was the interval between each wave containing the main questionnaire programme and the focus topics (see Topics and Questionnaires). Until wave four, panellists were invited each month. As a monthly invitation to a survey may be too burdensome for many respondents and could have a negative effect on their willingness to continue participation, starting with wave five, we increased the interval between each wave to two months.

We introduced a second level of frequency by dividing the respondents (who provided panel consent) from wave one into four groups. Starting in wave two, each group was invited in a different week of the month, that is, with one-week intervals between each group. With the increasing intervals between waves starting in wave five, we also increased the interval between the four groups in each wave. While waves two to four use a one-week interval between each group, waves five to seven use a two-week interval between each group. To integrate the refreshment sample into our design, we divided the refreshment participants into four groups before inviting them to wave five.

### Panel maintenance and incentives

The aims of the study were to launch the first wave quickly after the first contact restrictions (“lockdown”) in Germany, which were implemented in mid-March 2020. At that point, only the survey tool *keyingress*^7^ was available. As *keyingress* is not designed for panel surveys in terms of data management and providing incentives to respondents, we decided to change software. To this end, in waves three and four, respondents were invited to register on an online portal designed for the HOPP study and based on the software *panelingress*.^8^ All panellists who consented to be recontacted with a postal letter received an invitation to register themselves at the end of the wave four questionnaire. Overall, 3756 respondents registered themselves, that is, 1.9% of the individuals initially invited in wave one and 38.5% of wave one respondents who provided panel consent. Panellists who did not register themselves were not contacted in follow-up waves (see\*MERGEFORMAT Fig. 2).

Respondents in the refreshment sample were invited to register themselves at the end of the wave five survey. From wave five on, participants were invited to subsequent survey waves only if they successfully registered with their e-mail contact in the online portal. Overall, 4960 (61.2%) of refreshment respondents from wave five registered themselves. To motivate respondents to register themselves and to respond to future survey invitations, we provided incentives. For registering, participants received 500 points, the equivalent of a five-euro voucher. Participants could exchange their points for vouchers redeemable at various (online) shops, such as amazon.de, Thalia, Conrad, and Otto. We rewarded panellists with an additional 200 points for participation in each subsequent wave.

For all panellists, registration was a technical prerequisite to receive the promised voucher. Registered panellists could delete their registration at any time, e.g., even directly after receiving the voucher. However, this rarely occurred, as only 0.3% of the 8716 registered panellists withdrew their registration within a month after registration.

### Response rates across waves

Table 1 shows the response rates for all waves by panel status, differentiating between newly recruited respondents and returning panellists. For the sake of simplicity, we refer to wave one respondents who consented to be contacted for follow-up waves as *wave one panellists* and to respondents from the refreshment sample who registered themselves on the panel website as *refreshment panellists* hereafter. We calculated the response rates according to the AAPOR standard definitions for response rates using the definition for Response Rate 1 (RR1): the number of complete interviews by the number of invited cases (see AAPOR 2016). The response rates are based on complete interviews, defined as interviews in which respondents provided an answer to the last substantive question. If respondents provided their consent for administrative data linkage, we compared the age and gender between both data sources. We excluded cases for which age and gender did not match between the survey and administrative data (N_wave one_ = 265, N_refreshment sample_ = 283).

**Table 1:** Response rates (RR) for new recruits and panellists and realized number of analysis cases by wave

Wave	New recruits		Wave 1 panellists		Refreshment Panellists		Realized number of analysis cases	
	N_invited_	RR^a^	N_invited_	RR^a^	N_invited_	RR^a^	Overall	With record linkage consent
1	200,000	5.7	–	–	–	–	11,311	9548
2	–	–	9751	48.6	–	–	4746	4258
3	–	–	9751	41.7	–	–	4071	3673
4	–	–	9751	37.7	–	–	3682	3339
5	99,188	8.2	3739	79.5	–	–	11,072	9595
6	–	–	3744	82.4	4939	72.3	6659	6141
7	–	–	3737	80.6	4931	67.3	6334	5836

^a^AAPOR RR1

Of the 200,000 individuals invited in wave one, 5.7% responded to the survey (AAPOR RR1), and 4.9% initially consented to be contacted again (*wave one panellists*), resulting in 9751 *wave one panellists* who were invited to waves two to four. Of the respondents who gave panel consent, 5948 (61%) provided an e-mail address for further contact, whereas 3803 (39%) agreed to be contacted by mail. Table 1 shows a decreasing response rate from wave two (48.7%) to wave four (37.8%). We found that 65.6% of *wave one panellists* (N = 9751) completed at least one questionnaire in waves two to four, 21.2% of *wave 1 panellists* responded to all three waves, and 34.4% completed none of the follow-up waves.

In wave four, we moved our panel from *keyingress* to *panelingress* by inviting *wave one panellists* to register themselves on the panel website to continue their participation. Overall, 3755 *wave one panellists* registered themselves, that is, 1.9% of individuals initially invited to wave one and 38.5% of *wave one panellists*.

Among the refreshment sample of 99,188 individuals invited in wave five, 8.2% responded (AAPOR RR1), and 61.2% of refreshment respondents registered themselves on the panel website, that is, 5.0% of the refreshment sample. As we used conditional incentives in wave five and no incentives in wave one, we find it likely that the higher response rate in wave five can be attributed to using incentives. *Wave one panellists* who registered themselves in wave four (N = 3756) had a response rate of 79.5% in wave five.

For wave six and wave seven, we calculated the response rates separately for wave one and refreshment panellists. While *wave one panellists* had a response rate of 82.4% in wave and 80.6% in wave, refreshment panellists had a response rate of 72.3% in wave six and 67.2% in wave seven.

Table 1 also indicates the number of analysis cases by wave. In waves one and five, respondents were asked to provide informed consent for their individual survey responses to be linked to administrative datasets of the Federal Employment Agency (IEB) to enrich the survey data with administrative data.

The design of the HOPP study enables researchers to evaluate changes over time, using months and calendar weeks instead of data collection waves. We provide tables similar to Table 1 indicating the response rates and realized number of analysis cases by month (Table 3) and calendar weeks (Table 4) in the appendix.

## Analysis potential of the HOPP study

**Fig. 3:**
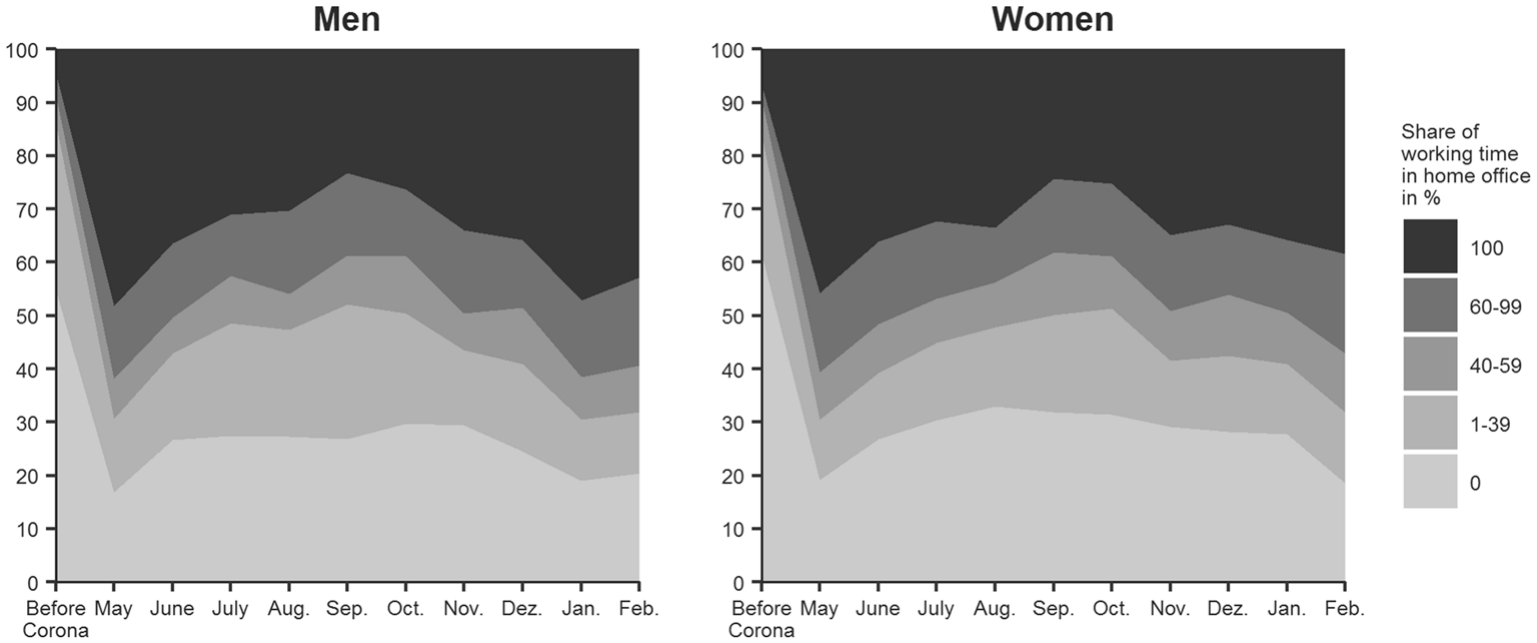
**Weekly working time in home office in relation to weekly total working time, shares of employed men and women, in percent (based on respondents who have the option to work from home)**. The number of cases differs by data collection month and ranges between 646 and 2457 for men and between 628 and 1971 for women. Values for “before corona” are based on the quotient of the answers to the two questions from wave 1 (May 2020): “Thinking about the time before the corona crisis, how many hours per week did your usual working hours consist of, including overtime worked, extra work, etc.?” and “Thinking back to before the corona crisis, how many hours a week did you regularly work from home before the crisis?”. Values for each month are based on the quotient of responses to the two questions, “Thinking about your last work week, how many hours did you work at home?” and “Thinking think about your last work week, how many hours did you actually work, including regular overtime, extra work, etc.?” The figure is weighted to represent individuals in Germany who had employment subject to social insurance contributions in 2018 and had the option of working at home during the data collection month (for more details on weights, see HOPP Data Manual: https://fdz.iab.de/de/FDZ_Individual_Data/HOPP.aspx)

The HOPP data enable researchers to track the development of various labour market-related indicators between May 2020 and February 2021. To show the analysis potential of the HOPP data, we use an updated analysis published in Frodermann et al (2021)^9^ showing the development of weekly time spent teleworking in relation to weekly total working time before the COVID-19 pandemic (see Fig. 3). Each month’s values in Fig. 1 include only individuals who self-reported having the option to work from home in a particular month. The share of individuals who have an option to work from home is 39% and does not change significantly across months. For the sake of simplicity, the values for each month are grouped into five categories ranging from 0, which is working solely at the workplace, to 100, which is working solely from home.

Figure 3 shows how the pandemic affected the share of time spent working from home in relation to the total working time for men and women. Before the pandemic, only 4% of men and 7% of women worked completely from home. At the beginning of the crisis, the share increases to 46% for men and 44% for women. From May to September 2020, the share of individuals decreases but stays higher than the *before corona* value (men: 26 %, women: 24%) and increases in the following month reaching a new peak in February 2021 (men: 45%, women: 38%). The shares of the other categories indicating that individuals work from home at least some of the time increase as well. For individuals who spent more than 40% of their working time teleworking, the share increases from 9% for men and women (before corona) to between 20 and 25% for men and between 18 and 30% for women. Compared to *before corona* (54% of men and 60% of women), a substantially lower share of men (16%) and women (19%) still worked completely from their workplace. Although this share increases again in June, it remains low during 2020 compared to the *before corona* value and decreases again in January and February 2021.

Preliminary HOPP data were used not only to assess the changes in teleworking time but also to address the effect on other labour market outcome variables, such as short time work, the subjective strain of employed parents with dependent children and the effects on the employment of older workers, reflecting HOPPs’ broad analytic potential.

Short-time work is an important measure of active labour market policy, especially in times of crisis, because it provides financial assistance for employers to prevent layoffs and secure jobs during economic downturns. Based on data from HOPP, Kruppe and Osiander (2020a, 2020b) published empirical findings on the use of short-time work during the early stage of the COVID-19 pandemic in Germany. These results are especially policy-relevant because official data from the FEA concerning short-time work are published with a 3-month time lag.

Fuchs-Schündeln and Stephan (2020) analyse the subjective strain of employed parents with dependent children. Three-quarters of working parents state that their workload increased during the pandemic. The proportion of women whose workload has increased sharply is higher than the proportion of men. Globisch and Osiander (2020) analyse how respondents who report being in a relationship share childcare responsibilities among themselves. Their results suggest that women continue to shoulder the greater part of childcare responsibilities. However, the proportion of men who assume more responsibility is increasing somewhat.

Westermeier (2020) focuses on the effects of the corona crisis on the employment of older workers. According to his results, the unemployment rate for older people is rising only moderately. However, they are particularly affected by the loss of marginal part-time employment (so-called “minijobs”). Older workers are less likely to work in home offices than younger colleagues. The reduction in working hours is only slightly greater in the 60+ age group than in the younger age groups.

## Data linkage and access

To enrich the survey data with administrative information on individuals, the data of those who gave consent are linked to administrative data available at the German Institute for Employment Research (IAB). This linkage expands research opportunities by including detailed records on earnings, labour market participation and unemployment or participation in active labour market policy measures at the daily level. In addition, the administrative data also provide several pieces of information on the characteristics of the firms where respondents work. Finally, the record linkage extends the observation period to 1975, the earliest year of administrative data availability. The name of the linked data product is HOPP-ADIAB. For each wave, the number of analysis cases with record linkage consent corresponds to 84% to 91% of the overall number of cases in the analysis sample (see the last column in Table 1).

The data of the IAB-HOPP-study are available to the international research community. After data collection, the data are subject to strict quality and data protection control and are disseminated to the research community from the Research Data Centre (FDZ) at the IAB. Three access modes are offered according to the degree of anonymization. The survey is available as Scientific Use Files (SUF) and can be analysed within the institutional environment of the researcher. The linked data are available only via remote execution via JoSuA (Eberle et al. 2017) or on site. Data access is free of charge; however, users are required to sign a Data Use Agreement with the FDZ and must comply with further requirements according to the access mode. Further information is available on the homepage of the FDZ, which also provides related survey documentation, e.g., a detailed description of the dataset and frequency tables (https://fdz.iab.de/).

## Future prospects

Currently, the HOPP study is conducting its eighth wave (April/May 2021) and is planning to conduct more waves for the duration of the corona crisis. Furthermore, there are plans to continue the HOPP study for at least 1 year after the crisis. However, the frequency of data collection will decrease, as we assume that most individuals have adapted to the situation.

The collected HOPP data presented in this paper can be combined with administrative data available at the IAB, which will be continuously updated. Combining HOPP and administrative data will enable researchers to evaluate the effects of the situation during the corona crisis on future employment biographies. Therefore, the analytic potential of the HOPP data will increase in the future. For instance, one research aim might be the evaluation of further education during the corona crisis on finding a job or improving one’s own job position. Another research question might be whether managing home-office and home schooling has a negative effect on parents’ careers.

Looking back to see ahead: our society has had three major crises during the last two decades (the financial crisis in 2008, the migration crisis in 2015 and the corona crisis in 2020), and the future crises that will impact the German labour market will certainly come. Especially as lifes become increasingly globally connected, economic crises everywhere can have a substantial impact on the life and work situations of people in Germany. As such, the HOPP is providing substantial data to assess changes in the labour market, understand the consequences of the current crisis and identify the need for policy action.

## Data Availability

The data access is described in the section Data Linkage and Access. Data and Code for specific analyses in this article is available at the Institute for Employment Research (IAB). Up-to-date access information can be found here: https://www.iab.de/en/daten.aspx

## Appendix

See Tables 2, 3, 4.

**Table 2:** Overview of variables up to wave 7

Variables	Wave						
	1	2	3	4	5	6	7
**Covid-19 situation**							
**Attitudes towards easing of Corona policy measures: **Opening public facilities/opening restaurants, cafés and bars/opening sports facilities/cancelling event ban of events up to 100 participants/cancelling general exit restrictions		X					
**Attitudes towards Corona policy measures: **Closure of schools, kindergartens/prohibiting private parties/closure of recreational, cultural facilities/closure of sports facilities/contact restrictions/waiver of private travel/prohibiting of spectators at professional sports/closure of bars, clubs, pubs/closure of restaurants, cafés/14-day quarantine in case of infection/wearing of masks outdoors/wearing of masks on public transport, stores/none of these measures appropriate						X	X
Informed about the Corona policy measures in force in my region						X	X
Germany-wide uniform Corona measures useful						X	X
**Social inclusion and democracy**							
Position in society							X
**Trust towards: **federal government/state government/political parties/Federal Constitutional Court/ press, media/social media/police/science and research/health policy							X
Political party preference							X
**Life satisfaction and worries**							
**Current situation: Worried about …** own health/health of relatives/financial situation/economic situation	X	X	X	X	X	X	X
**Satisfaction with …** health/sleep/free time/family life/contacts to friends and acquaintances/democracy in Germany/Crisis management of the government	X	X	X	X	X		X
General life satisfaction	X	X	X	X	X		X
Satisfaction with current professional activity						X	X
**Employment**							
**Current employment status: **employed (> 450 Euro per month)/employed (< 450 Euro per month)/self-employed *(wave 1-5)*, self-employed, with employees (*wave 6, 7*)/self-employed, without employee (*wave 6, 7*)/unemployed/housewife (husband)/maternity protection status, parental leave/partial retirement (“work phase”)/partial retirement (“release phase”)/retired, early retirement/school, vocational training, apprenticeship/student/federal voluntary service, voluntary military service/other	X	X	X	X	X	X	X
Contact frequency with people (not including colleagues) in professional activity						X	X
Restrictions in professional activity since March 2020						X	
Current restrictions in professional activity						X	X
**Employment lost: **No/employment (> 450 euros/month)/employment (< 450 Euro/month)/self-employment	X						
New employment found / employment restarted		X					
Worries about job loss		X	X	X		X	X
Current employment status (partner)		X	X	X	X	X	X
**Supervisor: **respects privacy/has understanding for family situation/supports me getting ahead professionally/is role model of how to be successful professionally and privately/knows how much work I do				X			
Worries about future career				X			
Concerns about opportunities regarding education, vocational or further training				X			
Employment with fixed-term contract						X	X
Employment at temporary employment agency						X	X
Main breadwinner: Current situation					X		
Main breadwinner: Before pandemic					X		
Supervisor function							X
Span of responsibility							X
**Norms in employer-employee relationship**							
**Own attitude towards norms: **avoiding short-time work in case of financial reserves of company/supplementing short-time work benefits in case of financial reserves in company/home office also for unfinished tasks (without children)/home office also for unfinished tasks (with children)/obligation to notify employees of where they are going on vacation							X
**Presumed attitude majority of employees: **avoiding short-time work in case of financial reserves of company/supplementing short-time work benefits in case of financial reserves in company/home office also for unfinished tasks (without children)/home office also for unfinished tasks (with children)/obligation to notify employees of where they are going on vacation							X
**Working hours **							
Working hours before Corona, per week	X				X		
Working hours last working week	X	X	X	X	X	X	X
Overtime last working week, hours						X	X
Partner’s working hours last working week			X	X	X	X	X
Possibility home office	X	X	X	X	X	X	X
Possibility home office (partner)			X	X	X	X	X
Working hours home office before Corona, per week	X				X		
Working hours home office last working week	X	X	X	X	X	X	X
Partner’s working hours home office last working week			X	X	X	X	X
Home office within normal working hours or during free time				X		X	
Home office before start of Corona crisis				X			
**Home office and work-life-balance**							
**No home office before pandemic because …** employer did not allow it/supervisors did not allow it/technical requirements were not met/preconditions at home were not given/professional activities from home are not possible/worsening of promotion prospects feared / presence important to superiors/wanted to separate work and privatelife/preferred working from home/other professional reasons/other private reasons				X			
**Current situation: Not working from home because … **employer does not allow it/supervisors do not allow it/technical requirements are not met/preconditions at home are not given/professional activities from home are not possible/other professional reasons/other private reasons				X		X	X
**Last working week: Not worked from home because … **employer did not allow it/supervisors did not allow it/technical requirements were not met/preconditions at home were not given/professional activities from home are not possible/worsening of promotion prospects feared/presence important to superiors/wanted to separate work and private life/preferred working from home/other professional reasons/other private reasons				X		X	X
**Previous experiences: Home office … **is burdensome/is stressful/is an enrichment/helps to manage tasks/helps to cope with work demands/helps to make better use of time/helps to balance work and personal life				X		X	
Preferences: How many days/week home office in the future				X			
**Work-life balance 1: **Private concerns make it difficult for me to concentrate on work/after work, lack of energy for private activities/miss out on leisure activities due to workload					X		
**Work-life balance 2: **Work demands interfere with private life/time demands of work make it difficult to meet private commitments/stress at work makes it difficult to meet private commitments/time spent on private demands leads to postponement of work/unfinished work due to family/partner demands/private life impaired by work obligations						X	
**Short-time work**							
Short-time work: Current receipt of short-time work benefits	X	X	X	X	X	X	X
Share of short-time work (in total working time)	X		X	X	X	X	X
Employer subsidy for short-time work benefits	X						
Employer subsidy (wording changed compared to wave 1)			X	X	X	X	X
Short-time work (partner): Current receipt of short-time work benefits			X	X	X	X	X
Short-time work since start of Corona crisis						X	X
**Abuse short-time work (crosswise 1):** Mother’s birthday in January/February & worked more than billing for short-time work						X	X
**Abuse of short-time work (crosswise 2):** Father’s birthday in January/February & same amount of work as before despite short-time work						X	X
**Abuse of short-time work (crosswise 3):** Mother’s birthday in a leap year & Short-time work although dismissal is announced						X	X
**Abuse of short-time work (direct)**: Worked more than short-time allowance/Same amount of work as before despite short-time work/Short-time work although dismissal is announced						X	X
**New professional/voluntary/other activities during short-time work: **No/employment (> 450 euros/month)/employment (< 450 euros/month)/self-employment/planning self-employment/further training/voluntary work/other		X			X		
**Financial benefits**							
**Currently receiving or recently applied for: **Unemployment benefits (“ALG”)/Means-tested basic income (“ALG II”)/housing benefit/other benefit	X						
**Already received before or since Corona crisis: **Unemployment benefits (“ALG”)/Means-tested basic income (“ALG II”)/housing benefit/other benefit	X						
**Further training**							
**Further training since begin of Corona crisis in March 2020: **Courses/Information events/Self-directed learning/learning opportunities during work/none			X				
Further training already planned or started before Corona crisis			X				
Planned further training not possible due to Corona crisis			X				
Further training: Reasons why participation possible despite Corona crisis			X				
Further training: Reasons why participation impossible due to Corona crisis			X				
Further training: Changed importance since Corona crisis			X				
Change of activities in current job			X				
**Job insecurity and consumer behavior**							
How much to spend from unexpected amount of money			X	X	X	X	X
Probability of unemployment in the next 3 months (employed)			X	X	X	X	X
Probability of finding a job in the next 3 months (unemployed)			X	X	X	X	X
**Household characteristics**							
Household size	X				X	X	
Living together with spouse/partner	X						
Number of children under 18 in household	X				X	X	
Monthly net household income	X			X	X		
Change in monthly net household income (compared to February 2020)	X				X		
**Household: Living (with) …** alone/spouse/partner/children under 18/other related persons/other non-related persons		X	(X)	(X)	X	(X)	(X)
**Year of birth of child 1–4 **(children under 18, living in household)		X	(X)	(X)	X	(X)	(X)
**Childcare and couples’ division of childcare and housework**							
Childcare (before Corona crisis): Full day		X					
Childcare (current situation): Full day		X			X	X	X
Childcare (before Corona crisis): Half-day		X					
Childcare (current situation): Half-day		X			X	X	X
Organization of childcare before the Corona crisis (respondents with partner)		X					
Organization of childcare current situation (respondents with partner)		X	X	X	X	X	X
Organization childcare before Corona crisis (respondents without partner)		X					
Organization of childcare current situation (respondents without partner)		X	X	X	X	X	X
Childcare: Change in burden due to Corona crisis	X						
Childcare: Change in time load in the last 2 weeks		X	X	X	X		
Childcare: Hours on average working day							X
**Emergency childcare during lockdown: **In March 2020/in April 2020/no emergency care					X		
**Couples’ division of housework (before Corona): **Housework/shopping, running errands/repairs/financial affairs, visits to authorities/childcare		X					
**Couples’ division of housework (current situation): **Housework/shopping, running errands/repairs/financial affairs, visits to authorities/childcare		X	X	X	X		
Care of relatives		X					
Care of relatives: Change in burden due to Corona crisis		X					
**Socio-demographic variables**							
Gender	X						
Year of birth	X						
Highest school degree	X						
Highest vocational degree	X						
University degree	X						
Born in Germany	X						
Year of arrival	X						
**Citizenship:** German/other EU country/non-EU country	X						
Parents born outside Germany	X						
Residence State						X	
Living space		X					
**Additional space at place of residence:** Balcony, terrace/yard/garden/none		X					
**Health**							
Health status last four weeks						X	X
**Frequency of feelings in last 4 weeks:** Angry/Anxious/Happy/Sad		X	X	X	X		
**Frequency of feelings of social isolation:** Company of others is missing/left out/socially isolated		X	X	X	X	X	X
**Feelings regarding childcare:** Tired, exhausted/overwhelmed/get along well/worried about children’s health		X			X		X
**Health during last 4 weeks: **depressed, gloomy/calm, balanced/a lot of energy/severe physical pain/accomplished less in everyday life due to physical health/less active at work due to health/accomplished less in everyday life due to mental health/less active at work due to health/limited social contact due to mental health						X	X

X question asked in respective wave, (X) question asked only if no data from previous wave available

**Table 3:** Response rates and analysis cases by month

Month	New recruits		Wave 1 Panellists		Refreshment panellists		Number of analysis cases	
	N_invited_	RR	N_invited_	RR	N_invited_	RR	Overall	With record linkage consent
May	200,000	5.7	–	–	–	–	11,311	9548
June	–	–	9756	45.0	–	–	4391	3943
July	–	–	9756	38.6	–	–	3860	3481
August	–	–	9756	35.9	–	–	3500	3175
September	49,594	7.3	1866	73.5	–	–	4972	4319
October	49,594	7.6	1892	79.7	–	–	5295	4595
November	–	–	1860	80.3	2395	68.9	3143	2923
December	–	–	1884	80.7	2543	71.2	3330	3051
January	–	–	1852	73.5	2388	58.5	2759	2575
February	–	–	1885	70.0	2553	56.1	2750	2520

**Table 4:** Response rates and analysis cases by calendar week

Wave	Month	Calendar week	New recruits		Wave 1 Panellists		Refreshment Panellists		Number of analysis cases	
			N_invited_	RR	N_invited_	RR	N_invited_	RR	Overall	With record linkage consent
1	May	19	200,000	3.9	–	–	–	–	7.702	6.484
2	June	23	–	–	2435	39.7	–	–	966	872
		24	–	–	2436	36.5	–	–	888	811
		25	–	–	2444	33.8	–	–	826	744
		26	–	–	2441	32.5	–	–	794	726
3	July	27	–	–	2435	31.2	–	–	760	698
		28	–	–	2436	31.0	–	–	754	684
		29	–	–	2444	27.5	–	–	671	602
		30	–	–	2441	29.1	–	–	710	647
4	August	31	–	–	2435	23.4	–	–	570	517
		32	–	–	2436	22.3	–	–	543	490
		33	–	–	2444	21.7	–	–	530	480
		34	–	–	2441	20.8	–	–	507	466
5	September	36	24,797	4.6	938	46.5	–	–	1575	1355
		38	24,797	4.4	928	35.2	–	–	1409	1232
	October	40	24,797	4.5	926	52.6	–	–	1615	1405
		42	24,797	4.8	966	56.6	–	–	1728	1504
6	November^a^	45	–	–	935	62.2	1171	48.1	1145	1064
		47	–	–	925	62.3	1224	48.5	1170	1093
	December	49	–	–	921	59.6	1308	49.1	1191	1085
		51	–	–	963	67.6	1235	54.9	1329	1216
7	January	2	–	–	933	64.1	1170	50.1	1184	1092
		4	–	–	919	67.2	1218	47.7	1199	1133
	February	6	–	–	919	61.0	1311	47.7	1187	1083
		8	–	–	960	62.7	1235	46.3	1174	1078

^a^Time of invitation changed from Friday to Monday.

## Footnotes

1 https://www.uni-mannheim.de/gip/das-gip/.

2 https://www.pairfam.de/.

3 https://www.diw.de/en/soep.

4 https://fdz.iab.de/de/FDZ_Individual_Data/HOPP.aspx.

5 Spell is the term the IEB uses to describe a reported labour market period.

6 The individual password expired after the participant used it for the first time to prohibit a person from participating in the survey more than once.

7 For more details on the software, see: https://www.ingress-survey.co.uk/Software/Survey-software-keyingress/.

8 For more details on the software, see: https://www.ingress-survey.co.uk/Software/Panel-software-panelingress/.

9 Note that our analyses may deviate, as Frodermann et al (2021) did not have access to the current HOPP data but used a pre-released dataset.
